# Functional lung avoidance in radiotherapy using optimisation of biologically effective dose with non-coplanar beam orientations

**DOI:** 10.1016/j.phro.2023.100518

**Published:** 2023-11-21

**Authors:** James L. Bedford, Merina Ahmed

**Affiliations:** The Institute of Cancer Research and The Royal Marsden NHS Foundation Trust, London, UK

**Keywords:** Carcinoma, Non-Small-Cell Lung, Retrospective Studies, Radiation Injuries, Tomography, Emission-Computed, Single-Photon, Perfusion

## Abstract

**Background and purpose:**

In external beam radiotherapy for non-small cell lung cancer, dose to functioning lung should be minimised to reduce lung morbidity. This study aimed to develop a method for avoiding beam delivery through functional lung and to quantify the possible benefit to the patients.

**Materials and methods:**

Twelve patients that were treated as part of a clinical trial of single photon emission computed tomography (SPECT) functional lung avoidance were retrospectively studied. During treatment planning, the dose in the lung was weighted by the relative intensity of the functional image. A single conformal beam was scanned systematically around the planning target volume to find optimum orientations and the resulting map of functional dose variation with gantry and couch angle was used to select five non-coplanar intensity-modulated beams, taking into account directions prohibited due to collision risk. Expected reduction in pneumonitis risk was calculated using a logistic model.

**Results:**

The volume of lung irradiated to a functionally weighted dose of 5 Gy was 11.8 % (range 3.5 %–22.0 %) for functional plans, versus 20.9 % (range 4.9 %–33.3 %) for conventional plans (*p* = 0.002). Mean functionally weighted dose was 4.1 Gy (range 1.3 Gy-7.2 Gy) for functional plans, versus 4.5 Gy (range 1.5 Gy-8.3 Gy) for conventional plans (*p* = 0.002). Predicted pneumonitis risk was reduced by 4.3 % (range 0.4 %-15.6 %) (*p* = 0.002).

**Conclusions:**

By seeking the optimum non-coplanar beam orientations, it is possible to reduce dose/volume lung parameters by 10% or more, consistently in all patients, regardless of the pattern of lung perfusion. A prediction model indicates that this will improve radiation-associated lung injury.

## Highlights

• Planned dose to lung was weighted according to lung perfusion to give biologically effective dose.
• A non-coplanar beam mapping technique was used to identify optimal beam orientations for avoiding functioning lung.
• Five intensity-modulated radiation therapy beams at the optimal orientations were able to reduce the functionally weighted lung dose by approximately 10%, giving a significantly reduced risk of radiation-associated lung injury.

## 1 Introduction

Over the last two decades, a number of studies have been conducted to incorporate lung function information into treatment planning of non-small cell lung cancer [1], [2]. The concept is to use a radiolabelled tracer in conjunction with a single photon emission computed tomography (SPECT) scan to define the perfused regions of the lung and this information is used in tandem with a planning computed tomography (CT) scan for treatment planning. The radiotherapy is delivered through the regions with poorer function, thereby leaving the more perfused or ventilated regions relatively undamaged, with the aim that the patients are less likely to suffer from radiation pneumonitis [3], [4], [5], [6], [7], [8]. A recent review therefore concludes that use of SPECT information for functional lung avoidance is valuable, although definite dose constraints for practical planning are deficient [9].

However, the clinical results have so far been disappointing [10], which may be due to several reasons. Firstly, definition of normally functioning lung is variable from study to study, as the threshold in the functional image is undecided. Typically, the threshold at which the lung is taken to be functioning is 30 % of the maximum SPECT signal, but there is no firm basis for this [11]. Secondly, directing radiation beams through regions of poor function is difficult in many patients due to the pattern of perfusion. If the perfusion deficits are broadly spread over the lung volume, it is difficult to direct beams through these regions [12], [13]. Thirdly, it is unclear how much sparing of functioning lung is necessary to produce a measurable clinical benefit.

This paper aims to address these difficulties. By using dose distributions weighted by the lung function, the need for a threshold in function is avoided. Most importantly, this study presents a method of selecting those non-coplanar beam orientations which direct radiation through the least functional parts of the lung, so that the benefit of functional lung avoidance is maximised. Finally, the relationship between mean lung dose and clinical rate of pneumonitis is used to investigate the expected clinical benefit resulting from the dosimetric lung sparing obtained.

## 2 Materials and methods

### 2.1 Patients and scans

In this study, 12 patients from a clinical trial aiming to correlate irradiated volumes of anatomic and functional lung with radiation induced lung damage [11] were examined retrospectively. The clinical trial itself was performed in accordance with the ethical standards of the institutional and/or national research committee and with the 1964 Helsinki declaration and its later amendments or comparable ethical standards. Written informed consent was obtained from all individual participants included in the study.

For radiotherapy treatment planning, the patients were CT scanned in breath-hold using an Active Breathing Coordinator (ABC) device (Elekta AB, Stockholm, Sweden) [14] and then SPECT scanned within four hours using 200 MBq of ^99m^Tc-radiolabelled macroalbumin aggregate (MAA), to determine lung function. Further details of the scanning protocol are given elsewhere [11]. The functional scans were rigidly registered with the CT scans using a series of surface markers which contained radiotracer for the SPECT scans and were also visible on the CT scans. Use of ABC for the planning CT scan, so as to optimise the quality of treatment plan, but free breathing for the SPECT scan, in view of the time taken to acquire the scan, resulted in poor image matching around the diaphragm, but none of the target volumes in this study were located in this area, so this was not considered to be an issue. Gross tumour volume (GTV) was delineated on the CT scans and an isotropic margin of 5 mm was added to define the clinical target volume (CTV). A further margin of 5 mm was then added to the CTV to create the planning target volume (PTV). For two patients treated without ABC, the PTV margin was 5 mm laterally and 10 mm superiorly and inferiorly.

### 2.2 Treatment planning

In this retrospective study, AutoBeam v6.1 [15], [16] was used to create functional inverse plans. The SPECT scans were renormalised to the maximum intensity value in the scan and the dose to the lung region was then weighted by the corresponding relative function [17]. To ensure that the PTV dose distribution was not affected by this process, the PTV plus a margin of 5 mm was excluded from the lung region with functional weighting. As the functional weighting was a continuous variable, it was not necessary to define thresholds for function. However, for purposes of comparison with other studies, the lung volumes with greater than 25 % function (FL_25%_) and 50 % function (FL_50%_) were delineated by thresholding.

Treatment planning was conducted using a beam model for the 6 MV beam of a Versa HD accelerator (Elekta AB, Stockholm, Sweden) [18]. To define the optimum beam orientations for delivery of functionally weighted dose, a single conformal beam was scanned systematically through all gantry angles from 180° on one side of the couch to 180° on the other side of the couch and, for each of these gantry angles, the beam was scanned from couch angle 270° to 90°, giving a grid of gantry/couch combinations. The beam was normalised so that the mean PTV dose was 1.0. The mean functionally weighted lung dose resulting from each beam orientation was then plotted. Orientations not feasible due to collisions of gantry and couch were then omitted.

Complete treatment plans were then constructed consisting of five static intensity-modulated radiation therapy (IMRT) beams, each containing 10 segments. The directions of these beams were manually chosen based on the maps of mean functionally weighted lung dose as a function of beam orientation. Generally, the orientations that minimised the functionally weighted mean lung dose were selected, but in some cases, it was necessary to choose orientations with higher lung dose in order to ensure homogeneous PTV coverage and conformal distribution of dose around the PTV. Final plans were calculated using a convolution algorithm [19], [20] on a dose grid of 2.5 mm × 2.5 mm × 2.5 mm.

For comparison, a standard treatment plan was constructed for each patient, consisting of a single anticlockwise volumetric modulated arc therapy (VMAT) arc from 179° to 181° gantry angle, with control points at 2° intervals. Similar to current clinical practice at this centre, the arcs were designed to be mostly conformally shaped so as to provide robust dosimetry in the event of tumour shrinkage during the course of treatment [21]. Functional dose weighting was initially turned off, so that the plan was based on physical lung dose. After optimisation, the functional weighting was applied for lung so that the plan could be compared with the functional treatment plan. To understand the potential bias of using IMRT for the functional treatment plans and VMAT for the comparison plans, a coplanar IMRT plan with five equally spaced beams was created, each beam having 10 segments per beam. As with the VMAT plan, optimisation was performed without function and then functional weighting was applied at the end.

### 2.3 Prediction of outcome

The expected clinical benefit of the functional planning approach was evaluated by calculating predicted radiation pneumonitis risk using the logistic model and fitting parameters described by QUANTEC [22]. For each patient and type of plan, the mean functionally weighted dose, *D_f_*, was applied to the formula so as to reflect the benefit of the biological optimisation. However, the process of calculating *D_f_* for each patient by weighting the physical dose by function and then taking the mean had the effect of rescaling the dose. On average, over the entire group of patients, this rescale factor was the mean relative function of the population, *k*. To remove the rescaling for application to the QUANTEC formula, the mean functional dose of each patient and plan was therefore divided by *k*. Then the probability of radiation pneumonitis, *p*, was given by:

(1) $$p=\frac{\exp\left(b_{0}+b_{1}D_{f}/k\right)}{1+\exp\left(b_{0}+b_{1}D_{f}/k\right)}$$

The fitting parameters were those given by Marks et al. [22]: *b*_0_ = -3.87 and *b*_1_ = 0.126 Gy^−1^. The value of *k* was estimated as part of the study.

The functional and standard plans were compared in SPSS v29 (IBM Corp., Armonk, NY) using two-tailed Wilcoxon matched pair signed-rank tests, with a null hypothesis that the statistics for the two types of plan were from the same distribution.

## 3 Results

### 3.1 Lung perfusion

**Fig. 1:**
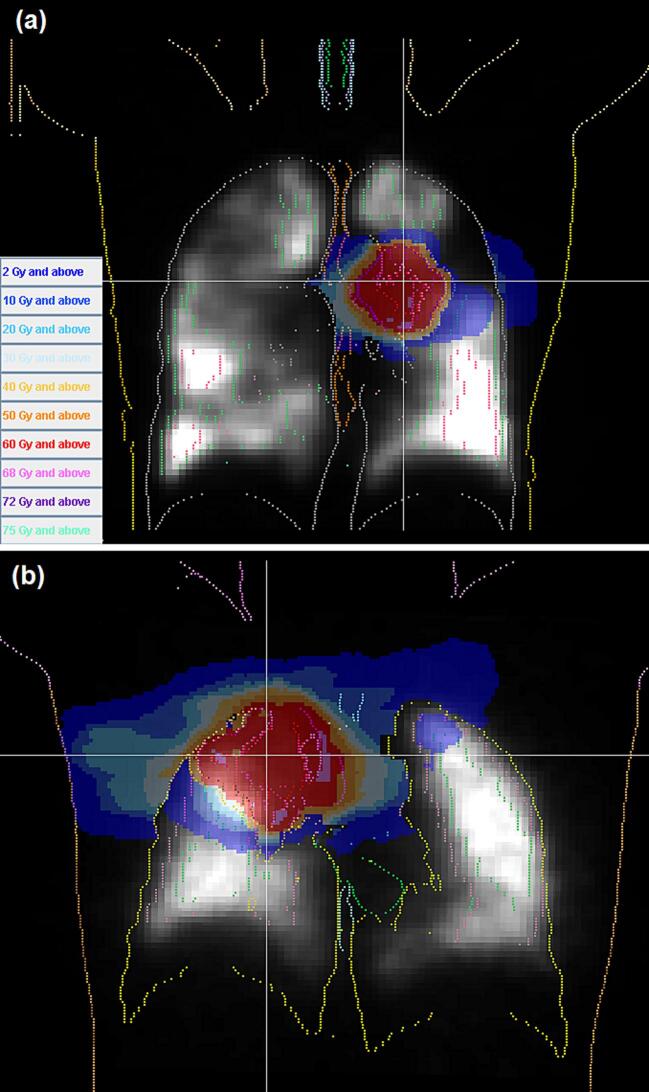
Coronal view of two patients, showing the relative lung perfusion. Patient (a) has variable perfusion, and patient (b) has more uniform perfusion. The dose distribution resulting from the functional non-coplanar treatment plan is shown in colour, with the colour key in the bottom left of (a).

Patterns of lung perfusion varied widely between patients. Some patients had moderate perfusion over the entire lung volume, whereas other patients had patches of well-perfused lung separated by regions of poor perfusion. Fig. 1 shows examples of each type of perfusion. After normalisation of the lung function, the mean function in the lung volume was found to vary among the patients from 0.18 to 0.38, with a median over the 12 patients of 0.24. Thus, the value of the function factor *k* in Eq. (1) was taken to be 0.24.

### 3.2 Beam orientation maps

**Fig. 2:**
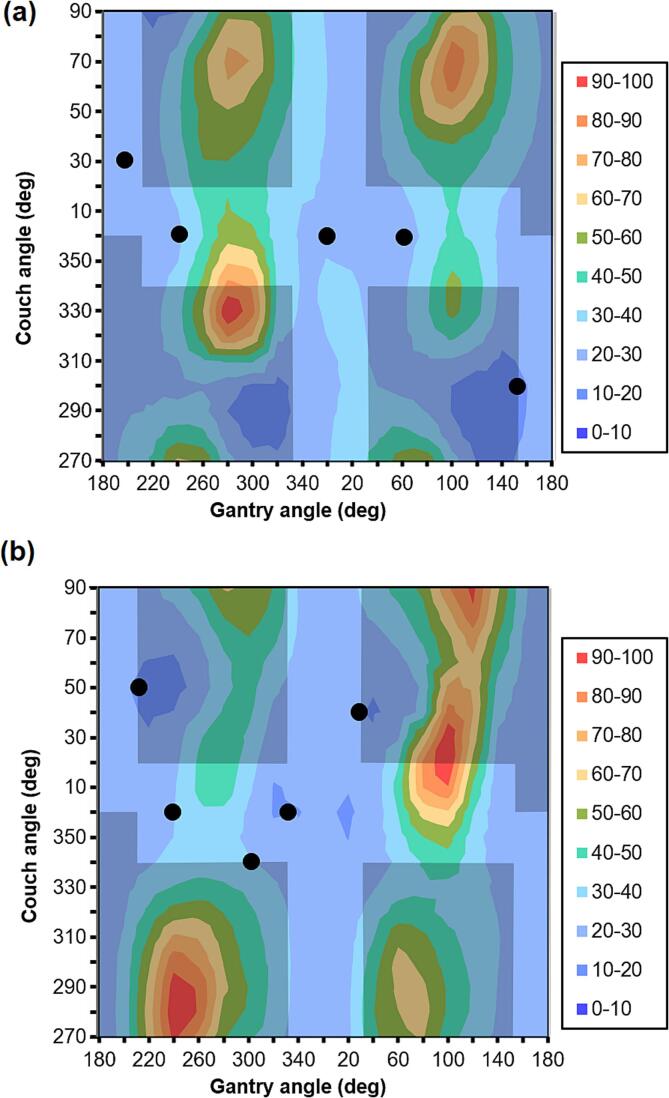
Beam orientation maps for the same patients as Fig. 1. The maps show the mean functionally weighted lung dose for a single beam at each combination of gantry and couch angle, for a 1 Gy mean dose to the planning target volume. The dose in each map is displayed as a percentage of the maximum dose in the map. The grey regions are collision zones and the black points are the selected beam orientations. Note that the left- and right-hand edges of the graph represent the same beam orientations (gantry 180°). Note also that gantry angle *g* at couch 90° is equivalent to gantry angle 360° - *g* at couch angle 270°. At gantry angle 0° and 180°, changing the couch angle merely changes the orientation of the collimator with respect to the PTV, so the vertical lines through gantry angles 0° and 180° are degenerate.

The beam orientation maps for the patients of Fig. 1 are shown in Fig. 2. Use of lateral beams generally gave a high mean functional dose as the beams traversed both lungs at their broadest dimension. Furthermore, with a large PTV, the beam aperture was larger, and consequently the mean lung dose was higher for all beam positions. For homogeneous perfusion, it was relatively difficult to find minima in which to place beams, but for inhomogeneous perfusion, there were several options available, in which case the greatest spread of beams was chosen so as to increase conformality.

### 3.3 Dose statistics

**Fig. 3:**
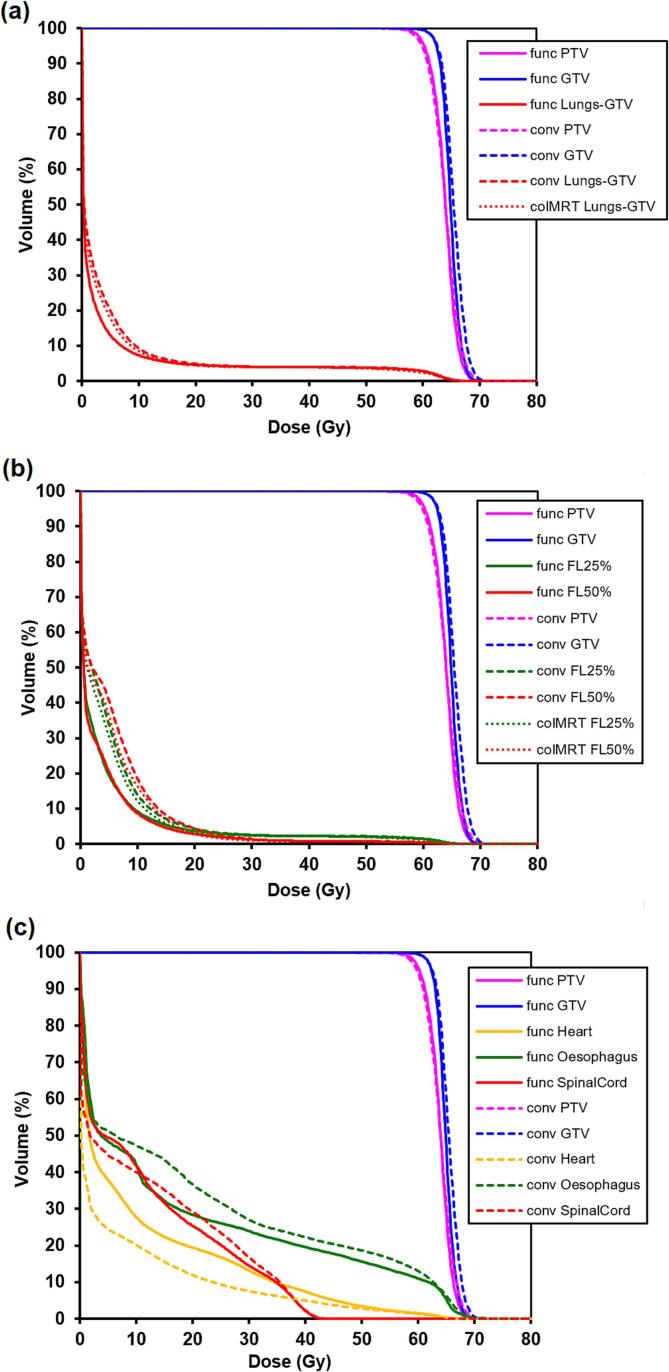
Mean dose-volume histograms for the functional (func) and conventional (conv) plans in the 12 patients. (a) Target volumes and whole lung, (b) target volumes and lung volume with at least 25 % (FL25%) function and at least 50 % (FL50%) function, (c) target volumes and normal structures. All of the lung dose-volume histograms show functionally weighted dose, whether conventionally or functionally planned. Parts (a) and (b) also show the dose-volume histograms for the coplanar IMRT comparison plan.

Fig. 3 shows mean dose-volume histograms for the functional and conventional plans. At functional doses above 20 Gy, there was no difference between the functional and standard plans because these higher doses related to the region immediately around the PTV, where it was not possible to minimise lung dose without compromising PTV coverage, and where functional weighting was also not applied. There was some lung sparing in the range of functionally weighted doses from 0 to 20 Gy. Note that with the median function being 0.24, a physical dose of 20 Gy equated to a functionally weighted dose of 5 Gy, where the difference between functional and conventional plans was greatest. The sparing of the thresholded FL_25%_ and FL_50%_ regions was shown to be considerable at doses of 0 to 20 Gy, again with maximum effect around 5 Gy, which was likely to be clinically important. Dose-volume histograms for the lung volumes with the coplanar IMRT plan are also shown in Fig. 3a and 3b. There was a small improvement in irradiated volume of lung with the IMRT plan compared to VMAT, but this did not detract from the obvious benefit of the non-coplanar functional plan.

**Fig. 4:**
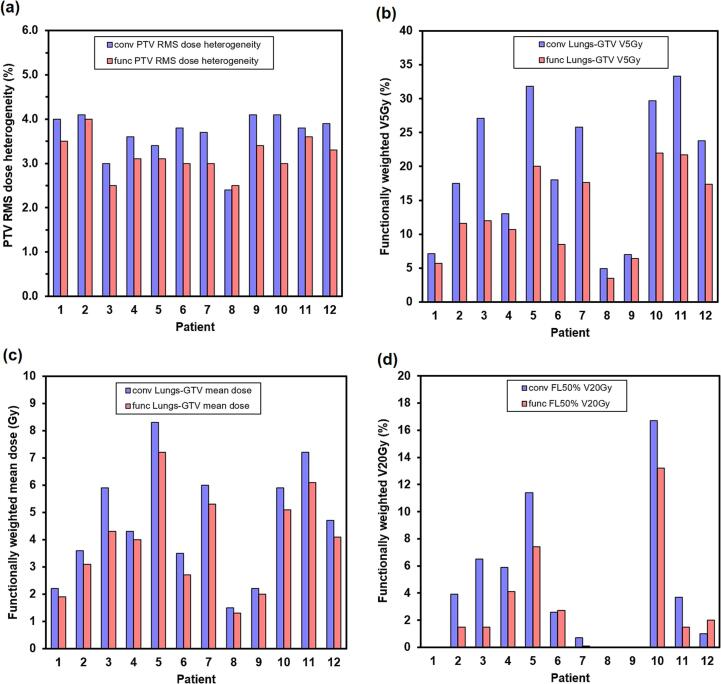
Comparison of conventional (conv) and functional (func) plans in the 12 patients. (a) Root mean square (RMS) dose heterogeneity with respect to 64 Gy. (b) Functionally weighted V_5Gy_. (c) Functionally weighted mean lung dose. (d) Functionally weighted V_20Gy_ for the lung with greater than 50 % function (FL50%). In (d), V_20Gy_ is shown, as opposed to V_5Gy_, as the function is higher in this region, so the functionally weighted dose is similar to the physical dose.

The statistics for the PTV and lung are shown for all of the patients individually in Fig. 4. The PTV homogeneity was generally better in the functionally weighted plans (*p* = 0.003), although this was unlikely to be clinically significant (Fig. 4a). The V_5Gy_ for the weighted lung dose was lower for the optimised plans (*p* = 0.002) (Fig. 4b). Similarly, mean functionally weighted lung dose was lower for all cases when using the orientation-optimised plans (*p* = 0.002) (Fig. 4c). In the well-functioning lung as defined by FL_50%_, there was also a lower functionally weighted V_20Gy_ (p = 0.03) (Fig. 4d).

**Fig. 5:**
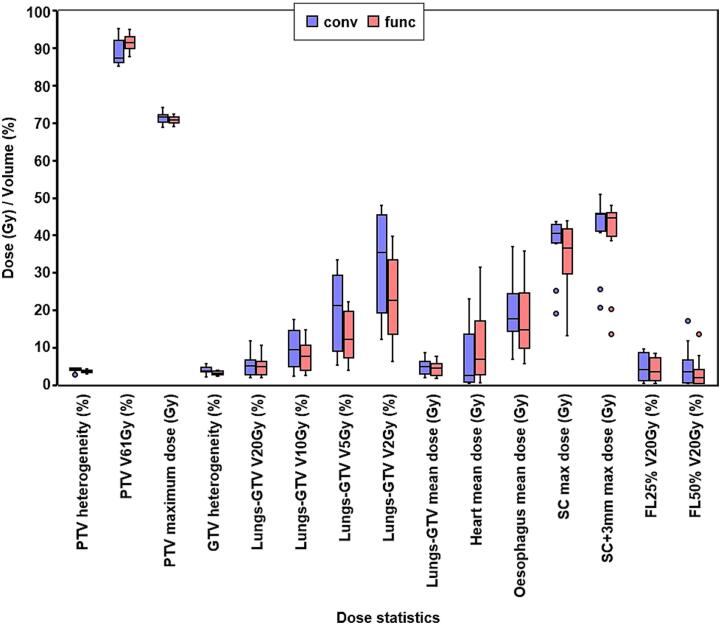
Comparison of key statistics for all structures for the conventional (conv) and functional (func) plans. The boxes show median and quartiles and the outliers are the points greater than 1.5 times the interquartile range from the quartile. PTV: planning target volume, GTV: gross tumour volume, SC: spinal cord, FL25%: lung with greater than 25 % function, FL50%: lung with greater than 50 % function. All statistics relating to lung (Lungs-GTV, FL25% and FL50%) are for functionally weighted dose and the remainder are for physical dose.

The statistics for all of the structures are shown in Fig. 5. Mean heart dose was slightly higher with the optimised plans (median 6.5 Gy; range 0.1 Gy to 31.2 Gy), than with coplanar plans (median 2.1 Gy; range 0.0 Gy to 22.8 Gy) due to the beams avoiding the lungs (*p =* 0.01). Mean dose to the oesophagus with the optimised plans (median 14.5 Gy, range 5.3 Gy to 35.6 Gy) was lower than with the standard coplanar plans (median 17.4 Gy, range 6.5 Gy to 36.8 Gy, *p =* 0.02). The spinal cord planning risk volume was similar between the two techniques (*p =* 0.1).

### 3.4 Prediction of outcome

**Fig. 6:**
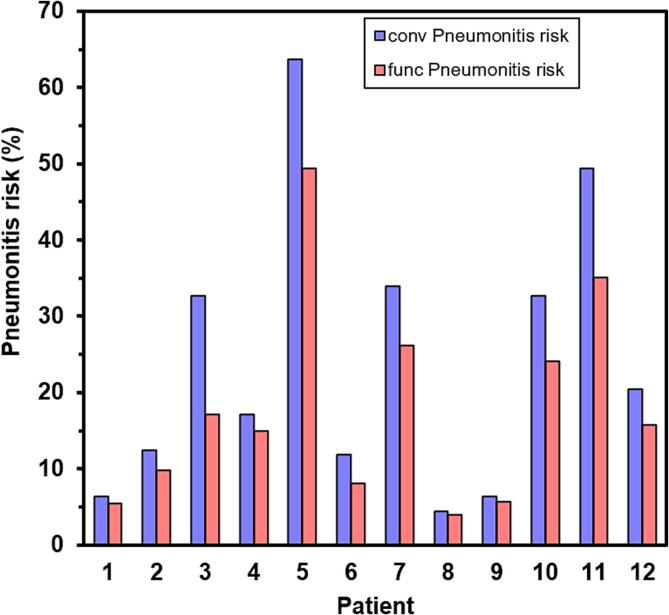
Comparison of predicted pneumonitis risk from the conventional (conv) and functional (func) plans in the 12 patients according to the QUANTEC logistic model.

The predicted radiation pneumonitis risk for the conventional and functional plans is shown in Fig. 6. The largest reductions in risk were seen in the patients with the highest initial risk, due to the increased steepness of the logistic model with increasing dose. However, some benefit was predicted for all patients. Overall, the median reduction in risk was 4.3 % (range 0.4 % to 15.6 %) (*p =* 0.002).

## 4 Discussion

In external beam radiotherapy for non-small cell lung cancer, dose to functioning lung should be minimised to reduce lung morbidity. This study weights the dose in lung by the relative function and then selects beam orientations to minimise that weighted lung dose, resulting in a reduction in irradiated volume from 21 % to 12 %, with a reduction in functionally weighted mean lung dose from 4.5 Gy to 4.1 Gy. This is predicted to reduce pneumonitis by approximately 4 %.

The study therefore supports the view of De Bari et al. [9] that use of SPECT to define functioning lung is a valuable asset in the treatment of non-small cell lung cancer. However, the production of treatment plans which significantly spare normal lung is found to require substantial application of functionally-weighted treatment planning and non-coplanar beam orientations. The use of functionally weighted dose enables the continuous spectrum of function values to be used, without having to make arbitrary decisions concerning the threshold level of function at which the lung is taken to be functioning normally [1], [3], [23], [24]. This type of weighting is very simple to apply, although such an option is not currently available in many treatment planning systems, so widespread implementation would require input from the vendors. The limitation of using ABC for the planning CT scan and free breathing for the SPECT could be resolved in a future study by the use of deformable image registration to adapt the SPECT scan to the contour of the breath-hold planning scan.

The use of non-coplanar beams, although not as streamlined practically as a single VMAT arc, is necessary to maximise the avoidance of functioning lung, and in today’s complex treatment environment, is actually a relatively simple technique. Several non-coplanar VMAT arcs could be used in place of the fixed IMRT beams, but as the IMRT beams are directed very specifically at the minima of the beam orientation maps, it is expected that spreading out the orientations over VMAT arc lengths would reduce the value of the solution, so is not presently recommended. Adding an automated selection algorithm to the present study is expected to increase the convenience of the beam selection in the event of clinical application, but it is not expected to improve the dosimetric or clinical benefit of the technique.

Taking the functional weighting factor, *k* (equation (1)) to be approximately 0.24, the observed reduction in functional mean lung dose corresponds to a conventional physical mean dose of 18 Gy, reduced to 16 Gy. Similarly, with functional V_5Gy_, which approximately translates to V_20Gy_ in conventional physical absorbed dose, the value can be reduced by 40 % of its initial value. These benefits in functional lung dose are similar those reported previously, although the deliverability of some of the previous techniques is unclear [24], [25]. Both mean lung dose and irradiated volume are in some way related to the clinical incidence of radiation pneumonitis [22], [26], [27] and dosimetric statistics of the functioning subvolumes of lung are also known to be correlated with post-radiotherapy lung function outcomes [11], [28]. With these results, it is expected that a clinical trial similar to that reported by Yaremko et al [10] would report a benefit for functional lung avoidance. Vinogradskiy et al. [29] and Miller et al. [30] report positive results in a phase II non-randomised trial against historical controls for patients selected according to considerable functional deficits around the PTV. The dosimetric benefits of the present study would enable trials such as these to be opened to a wider spectrum of patients. Note, however, that some of these reported studies are based on ventilation rather than perfusion and the relationship between the two varies considerably [31]. Both ventilation and perfusion should be correlated for lung function to be effective [32], so it is possible that both of these should be included in clinical studies [33].

Some of the studies in the literature show an increase in PTV dose inhomogeneity and an increase in heart dose, due to the redistribution of beams through the mediastinum to avoid functioning lung [7], [24]. In the present study, no attempt is made to force the critical structures to receive equal dose, so the change of beam orientations has a noticeable effect on the organs at risk, including the heart. The increase in mean heart dose from 2.1 Gy to 6.5 Gy may be of relevance for long term cardiac morbidity, but 6.5 Gy is still substantially lower than the 10 Gy reported by Vinogradskiy et al. [29] and the 13 Gy reported by Yaremko et al. [10]. It is also much lower than the constraint of median dose less than 25 Gy suggested by Khalil et al. [34] in a proposed study protocol. A possible means of avoiding a higher heart dose is to add the mean heart dose to the mean functional lung dose when creating the beam orientation maps, or to generalise the method further to include all organs at risk with contributions weighted by importance factors, so that the beam orientation maps represent objective function maps [35]. The entire beam selection method may also be used for avoidance of other parallel organs.

The pneumonitis risk model calculated that the reduction in dose to functioning lung demonstrated in this study should translate into a clinical reduction of pneumonitis by around 4 %, although in some patients with high initial lung doses due to large PTV, the reduction in risk of lung damage was as much as 20 %. The greatest benefit was expected to be observed in patients with unevenly perfused lungs and in patients having focal mismatch defects. The value of the method was also expected to be maximal for patients with previous lung surgery where dose could be delivered towards *peri*-operative cavities and in patients with upper lobe tumours where better cardiac sparing could be achieved. However, the clinical performance of the technique could ultimately only be quantified by a well-controlled clinical trial.

## CRediT authorship contribution statement

**James L. Bedford:** Conceptualization, Methodology, Software, Formal analysis, Writing – original draft. **Merina Ahmed:** Methodology, Investigation, Writing – review & editing.

## Declaration of Competing Interest

The authors declare the following financial interests/personal relationships which may be considered as potential competing interests: JB: research agreement with Accuray Inc. MA: research funding from BMS and MSD, advisory fees from AstraZeneca and BMS, lecture fees from AstraZeneca.
